# Diagnostic Performance of the FilmArray BCID2 Multiplex PCR Panel Applied to Excised Heart Valve Specimens from Patients with Infective Endocarditis

**DOI:** 10.3390/microorganisms14102411

**Published:** 2026-10-21

**Authors:** Agata Bilewska, Joanna Skrzeczyńska, Monika Dorota, Anna Skowrońska, Elwira Wojtyniak, Katarzyna Darmofalska, Agnieszka Woźniak, Kamil Borek, Beata Sulik-Tyszka, Grażyna Skolimowska, Krzysztof Wróbel, Wojciech Michalski, Agnieszka Magdziak

**Affiliations:** 1 Department of Cardiac Surgery, Medicover Hospital, 02-972 Warsaw, Poland; agatabilewska81@gmail.com; 2 Faculty of Medicine, Lazarski University, 02-662 Warsaw, Poland; 3 Department of Clinical Microbiology, Maria Skłodowska-Curie National Research Institute of Oncology, 02-781 Warsaw, Poland; joanna.skrzeczynska@nio.gov.pl (J.S.); monika.dorota@nio.gov.pl (M.D.); anna.skowronska@nio.gov.pl (A.S.); elwira.wojtyniak@nio.gov.pl (E.W.); katarzyna.darmofalska@nio.gov.pl (K.D.); agnieszka.wozniak@nio.gov.pl (A.W.); kamil.borek@nio.gov.pl (K.B.); beata.sulik-tyszka@nio.gov.pl (B.S.-T.); agnieszka.magdziak@nio.gov.pl (A.M.); 4 Medicover Hospital, 02-972 Warsaw, Poland; grazyna.skolimowska@medicover.pl; 5 Digital Medicine Center, Maria Skłodowska-Curie National Research Institute of Oncology, 02-781 Warsaw, Poland; wojciech.michalski@nio.gov.pl

**Keywords:** BCID2 panel, heart valves, infective endocarditis

## Abstract

Infective endocarditis (IE) is a life-threatening disease in which rapid identification of the causative pathogen is essential for appropriate antimicrobial therapy. Although blood cultures remain the standard microbiological method, complementary molecular techniques have the potential to improve pathogen detection in selected clinical settings. This study evaluated the diagnostic performance of the FilmArray Blood Culture Identification 2 (BCID2) multiplex polymerase chain reaction (PCR) panel applied off-label to excised heart valve specimens. Excised heart valve specimens obtained from 47 patients undergoing surgery for IE were analysed using the BCID2 panel. The BCID2 results were compared with blood and valve culture results. Forty-two patients were included for blood cultures and 46 for valve cultures. Using blood culture as the clinical reference method, the BCID2 assay demonstrated a sensitivity of 78.4% (95% confidence interval (CI): 61.8–90.2%), a specificity of 60.0% (95% CI: 14.7–94.7%), a positive predictive value (PPV) of 93.5% (95% CI: 78.6–99.2%), and a negative predictive value (NPV) of 27.3% (95% CI: 6.0–61.0%). Agreement between the BCID2 assay and blood culture was low (Cohen’s κ = 0.253; 95% CI: −0.068–0.573; *p* = 0.067). In the secondary comparison using conventional valve culture, sensitivity was 8.8% (95% CI: 1.9–23.7%), whereas specificity was 100% (95% CI: 75.3–100%). These findings suggest that off-label application of the BCID2 panel to excised heart valve specimens may complement conventional microbiological methods in selected patients with infective endocarditis. However, a negative BCID2 result should not be used to exclude infective endocarditis, and larger multicentre studies are warranted to further evaluate the clinical utility of this approach.

## 1. Introduction

Infective endocarditis (IE) remains a serious clinical condition associated with substantial morbidity and mortality despite continuous advances in diagnostic and therapeutic strategies. It is caused by microorganisms entering the bloodstream and colonising the endocardium, most commonly the heart valves. Major predisposing factors include previous infective endocarditis, prosthetic heart valves or valve repair material, congenital heart disease, intravenous drug use, and the presence of intracardiac prosthetic devices [1,2].

The diagnosis of IE is often challenging because its clinical presentation is highly variable. Although fever, a new heart murmur, and positive blood cultures represent the classical diagnostic triad, these findings are frequently incomplete or absent, particularly in older adults and patients with significant comorbidities. Consequently, the diagnosis relies on the integration of clinical, microbiological, and imaging findings according to established diagnostic criteria [3,4]. The clinical manifestation can often mimic neurological, rheumatologic, surgical, or dermatological conditions, complicating the diagnosis. Echocardiography and blood cultures remain the cornerstone of the diagnostic work-up [1,2,5].

Recent advances in molecular microbiology have created new opportunities for the rapid detection of microorganisms directly from clinical specimens. Unlike conventional culture-based methods, molecular assays can provide microbiological results within a short time after specimen processing and may complement routine diagnostic techniques in selected clinical settings. However, their diagnostic performance when applied directly to excised heart valve tissue has not yet been fully established [2,3].

The FilmArray Blood Culture Identification 2 (BCID2) panel (bioMérieux) is a fully automated multiplex polymerase chain reaction (PCR) assay designed for the identification of bacterial and fungal pathogens, together with selected antimicrobial resistance genes, from positive blood culture. In the present study, the assay was applied off-label to homogenized excised heart valve specimens.

The aim of this prospective study was to assess the feasibility and microbiological concordance of the FilmArray BCID2 panel applied off-label to excised heart valve specimens, using blood culture as the clinical reference method.

## 2. Materials and Methods

### 2.1. Clinical Specimens

The study included consecutive aortic, mitral, and tricuspid valve specimens excised during cardiac surgery performed at Medicover Hospital (Warsaw, Poland) in patients with a clinical diagnosis of infective endocarditis during the predefined study period. Immediately after excision, each specimen was placed in a sterile container with 1–2 drops of sterile 0.9% saline and transported at room temperature (15–26 °C) to the Department of Clinical Microbiology, Maria Skłodowska-Curie National Institute of Oncology, Warsaw, Poland.

### 2.2. Valve Specimen Processing

Valve processing was performed in a Class II biological safety cabinet. The excised valve fragment was placed in a sterile mortar, and 2 mL of sterile 0.9% saline was added. The specimen was homogenized for 5–10 min, followed by the addition of a further 2 mL of sterile saline. After thorough mixing, the homogenate was transferred to a sterile 5-mL tube and allowed to stand for 5–7 min to obtain a supernatant.

An aliquot of 200 µL of the supernatant was loaded into the BCID2 pouch. Thereafter, the fully automated FilmArray BCID2 assay (Bio-Fire FilmArray Torch System, bioMerieux, France), integrating nucleic acid extraction, amplification, and detection in a closed system, was performed. Results were available after approximately 70 min. The total processing time, including specimen registration, valve homogenisation, and PCR analysis, was approximately 1.5 h. Because the BCID2 panel is validated for use with positive blood cultures, it was used off-label in our study.

The BCID2 panel detects the following microorganisms: *Enterococcus faecalis*, *Enterococcus faecium*, *Listeria monocytogenes*, *Staphylococcus* spp., *Staphylococcus aureus*, *Staphylococcus epidermidis*, *Staphylococcus lugdunensis*, *Streptococcus* spp., *Streptococcus agalactiae*, *Streptococcus pneumoniae*, *Streptococcus pyogenes*, *Acinetobacter calcoaceticus-baumannii complex*, *Bacteroides fragilis*, *Enterobacterales*, *Enterobacter cloacae complex*, *Escherichia coli*, *Klebsiella aerogenes*, *Klebsiella oxytoca*, *Klebsiella pneumoniae group*, *Proteus*, *Salmonella* spp., *Serratia marcescens*, *Haemophilus influenzae*, *Neisseria meningitidis*, *Pseudomonas aeruginosa*, *Stenotrophomonas maltophilia*, *Candida albicans*, *Candida auris*, *Candida glabrata*, *Candida krusei*, *Candida parapsilosis*, *Candida tropicalis*, *Cryptococcus neoformans/gattii*. It also detects the following resistance genes: CPEs (Carbapenemase-producing *Enterobacterales*)—IMP, KPC, NDM, OXA-48-like, and VIM; ESBL (extended spectrum betalactamase)—CTX-M; MRS (methicillin-resistant *Staphylococcus*)—*mecA/C*, *mecA/C*, and MREJ (MRSA); VRE (vancomycin-resistant *Enterococcus*)—*vanA/B*; and colistin resistance—*mcr-1*.

### 2.3. Blood Culture Processing

Blood cultures were performed before cardiac surgery at the institutions where the patients received clinical care. Because patients were treated at multiple hospitals, blood culture analyses were performed in different microbiology laboratories. Information on blood culture results and the microorganisms identified was retrieved retrospectively from the patients’ medical records. For the purposes of this study, blood culture served as the clinical reference method for comparison with the BCID2 assay, as no universally accepted reference standard is available for evaluating molecular testing performed on excised heart valve specimens.

### 2.4. Valve Culture

Valve specimens were cultured in the external microbiology laboratory providing diagnostic services for Medicover Hospital using the laboratory’s routine microbiological procedures. Species identification was performed by matrix-assisted laser desorption/ionization time-of-flight mass spectrometry (MALDI-TOF MS) using the MALDI Bio-typer system (Bruker, Bremen, Germany). Valve culture results and species identification were retrieved from the patients’ medical records.

### 2.5. Ethical Approval

The study protocol was reviewed by the Bioethics Committee, which issued a positive opinion regarding the analysis of surgically removed heart valves (approval No. KB-078-14/24).

### 2.6. Statistical Methods

The primary analysis assessed the ability of BCID2 to detect microbiological positivity, using blood culture as the clinical reference method. Sensitivity, specificity, positive predictive value (PPV), negative predictive value (NPV), and accuracy were calculated based on the binary positive/negative results of the two methods. These measures were used to describe the performance of BCID2 for detection of microbiological positivity relative to the clinical reference method and were not intended to represent definitive diagnostic accuracy for infective endocarditis or definitive accuracy of organism identification. A secondary comparison assessed the binary positive/negative results of BCID2 and valve culture. Sensitivity, specificity, positive predictive value (PPV), negative predictive value (NPV), and accuracy were calculated using the same approach. As a secondary exploratory analysis, concordance of microorganism identification between BCID2 and blood culture was assessed among patients with positive blood cultures at the genus and species levels. Genus-level concordance was defined as detection of organisms belonging to the same genus by both methods, whereas species-level concordance was defined as detection of the same species by both methods. In patients with polymicrobial results, concordance was considered present when at least one microorganism was shared between the two methods. Exact 95% confidence intervals (95% CI) for sensitivity, specificity, PPV, NPV, and accuracy were calculated using the Clopper–Pearson method. Agreement between binary BCID2 and blood culture results, as well as between binary BCID2 and valve culture results, was additionally assessed valuated using Cohen’s kappa coefficient (κ), with the corresponding 95% CI. Statistical analyses were performed using IBM SPSS Statistics, Version 23.0 (IBM Corp., Armonk, NY, USA).

## 3. Results

### 3.1. Study Population

Between May 2023 and December 2025, a total of 50 BCID2 assays were performed on excised heart valve specimens (aortic, mitral, tricuspid; native, prosthetic valves) obtained from 47 patients with infective endocarditis who underwent valve surgery. The study population comprised 35 (74.5%) men and 12 (25.5%) women, with a median age of 55 years (range, 21–82 years). In three patients, multiplex PCR testing was performed separately on two valves. Because identical results were obtained from both valves, only one result per patient was included in the statistical analysis.

Forty-two patients were included in the statistical analysis after excluding five cases with missing blood culture results from referring centers. Valve culture results were available for 46 patients.

The median time between blood culture and surgery was 13 days (range 0–50).

### 3.2. Patient-Level Microbiological Findings

Patient-level microbiological findings are summarized in Table 1. Using BCID2, microorganisms were detected in 35 of 47 patients (74.5%). The most frequently detected target was *Streptococcus* spp., identified in 16 patients (34%). *Streptococcus pneumoniae* and *Streptococcus agalactiae* were each detected in one patient (2.15%). The next most frequently detected targets were MSSA, detected in 10 patients (21.3 *Enterococcus faecalis* in 6 patients (12.8%), and MSSE in 2 patients (4.3%). Concurrent detection of MSSA and *Enterococcus faecalis* was observed in one patient (2.1%). No antimicrobial resistance genes included in the BCID2 panel were detected. BCID2 results were negative in 12 patients (25.5%). Blood culture results were available for 42 patients. Among these, blood culture was positive in 37 patients and negative in 5 patients. Detailed patient-level blood culture results are presented in Table 1.

### 3.3. Comparison of BCID2 with Blood Culture

The comparison between BCID2 and blood culture results is presented in Table 2. Among the 42 patients with available blood culture results, BCID2 and blood culture showed concordant microbiological positivity in 32 cases (76.2%). This included 29 patients with positive results by both methods and 3 patients with negative results by both methods. Discordant microbiological positivity was observed in 10 cases, including 2 BCID2-positive/blood culture-negative cases and 8 BCID2-negative/blood culture-positive cases.

Using blood culture as the clinical reference method the sensitivity of BCID2 for detection of microbiological positivity was 78.4% (29/37; 95% CI, 61.8–90.2%), while specificity was 60.0% (3/5; 95% CI, 14.7–94.7%). The PPV was 93.5% (29/31; 95% CI, 78.6–99.2%), the NPV was 27.3% (3/11; 95% CI, 6.0–61.0%), and accuracy was 76.2% (32/42; 95% CI, 60.5–87.9%). Cohen’s kappa coefficient was 0.253 (95% CI, −0.068–0.573; *p* = 0.067). Among the 37 patients with positive blood cultures, concordance at the genus level was observed in 26/37 patients (70.3%), whereas species-level concordance was observed in 16/37 patients (43.2%) (Table 3).

**Table 3:** Diagnostic performance of BCID2 for detection of microbiological positivity using blood culture as the clinical reference method.

95% CI	Estimate	Measure
61.8–90.2%	78.4% (29/37)	Sensitivity
14.7–94.7%	60.0% (3/5)	Specificity
78.6–99.2%	93.5% (29/31)	PPV
6.0–61.0%	27.3% (3/11)	NPV
60.5–87.9%	76.2% (32/42)	Accuracy
−0.068–0.573	0.253	Kappa

### 3.4. Comparison of Valve Culture with BCID2

The comparison between valve culture and BCID2 results is presented in Table 4. Among the 46 patients with available valve culture results, both methods yielded positive results in 3 cases, whereas both methods yielded negative results in 12 cases. Discordant microbiological positivity was observed in 31 cases, all of which were BCID2-positive and valve culture-negative.

Diagnostic performance measures for valve culture compared with BCID2 are summarised in Table 5. Sensitivity was 8.8% (3/34), specificity was 100.0% (12/12), the PPV was 100.0% (3/3), the NPV was 27.9% (12/43), and overall accuracy was 32.6% (15/46). Corresponding 95% confidence intervals are presented in Table 5. Agreement between valve culture and BCID2, assessed using Cohen’s kappa coefficient, was 0.048 (95% CI: −0.011 to 0.107; *p* = 0.287).

Among the three patients with positive valve cultures, genus-level concordance was observed in 3/3 cases (100.0%), whereas species-level concordance was observed in 2/3 cases (66.7%).

## 4. Discussion

Infective endocarditis remains a diagnostic challenge due to its heterogeneous clinical presentation. IE should be considered in all patients with sepsis or fever of unknown origin in the presence of predisposing risk factors [2]. Positive blood cultures remain the cornerstone of IE diagnosis [3]. The average turnaround time for blood culture results ranges from 3 to 7 days, depending on the growth characteristics of the microorganisms and the methods used for their identification and antimicrobial susceptibility testing [4]. Technological advances in microbiology, particularly the introduction of molecular methods, have revolutionised diagnostics by substantially reducing the time required to obtain results.

This study evaluated the off-label application of the FilmArray BCID2 assay for detecting microorganisms in excised heart valve specimens from patients with infective endocarditis. BCID2 demonstrated a sensitivity of 78.4% and a high positive predictive value (93.5%) for the detection of microbiological positivity compared with blood culture, with an overall agreement of 76.2% between the two methods. In addition, the assay detected microorganisms in numerous valve specimens that remained culture-negative, whereas conventional valve culture showed very low sensitivity, consistent with its limited diagnostic yield in excised heart valve tissue.

The combination of a high positive predictive value and a low negative predictive value indicates that a positive BCID2 result may provide clinically meaningful microbiological evidence when a pathogen included in the assay panel is detected, whereas a negative result should not be interpreted as excluding infective endocarditis. This limitation reflects both the restricted range of microorganisms included in the assay panel and the moderate sensitivity observed in this study. Accordingly, BCID2 should be regarded as an adjunct to, rather than a replacement for, established microbiological and clinical diagnostic methods.

The results obtained, particularly regarding specificity and PPV for the detection of microbiological positivity, differ slightly from those reported in the literature.

Published studies have reported a wide range of sensitivity, specificity, PPV, and NPV values for molecular diagnostic methods, depending on the assay used and the selected reference standard [5]. Breitkopf et al. [6], using PCR and sequencing, showed a sensitivity of 41.2%, specificity of 100%, PPV of 100%, and NPV of 26.2% relative to valve culture. Leli et al. [7] compared the multiplex SeptiFast assay with valve culture and observed a sensitivity of 95%, specificity of 100%, PPV of 100%, and NPV of 83.3%. Similarly, Fernández et al. [8] demonstrated 100% sensitivity for the SeptiFast assay compared with blood culture, valve culture, and SeptiFast tests performed on blood culture material. The highest sensitivity for valve PCR (96%) was noted by Marín et al. [9].

In 28 cases, the BCID2 results obtained from valve samples were consistent with blood culture. Importantly, the absence of genes associated with resistance to methicillin in *Staphylococcus aureus* and resistance to vancomycin in *Enterococcus faecalis* can help antimicrobial therapy.

Additionally, results from valve testing using the BCID2 panel, which detected *Staphylococcus epidermidis* (2 cases), confirmed that the strain cultured from the blood was not a contaminant, but rather the true pathogen causing IE.

Only three cases gave negative results by both methods (blood culture and BCID2). Blood cultures were negative in 5 of 42 patients (11.9%), whereas BCID2 results were negative in 12 of 47 patients (25.5%). According to published data, blood cultures remain negative in 2.5% to 31% of patients with suspected IE due to prior antimicrobial therapy, low microbial burden, the presence of fastidious and slow-growing organisms such as the HACEK group (*Haemophilus* spp., *Aggregatibacter actinomycetemcomitans*, *Cardiobacterium hominis*, *Eikenella corrodens*, and *Kingella kingae*), or pathogens that cannot be cultured in routine media, including *Coxiella burnetii*, *Bartonella* spp., *Mycoplasma* spp., *Chlamydia* spp., and *Tropheryma whipplei* [5,10].

Previous studies have consistently shown that molecular methods improve pathogen detection in excised heart valve tissue compared with conventional valve culture, particularly in patients receiving prior antimicrobial therapy or presenting with culture-negative infective endocarditis [6,7,8,9,10]. Similar findings have been reported using broad-range PCR, multiplex PCR, and syndromic molecular panels, indicating that the diagnostic benefit is not restricted to a single molecular platform [6,7,8,9,10,11,12]. The study by Bast et al. [11] provides the closest methodological precedent, demonstrating the feasibility of applying the original FilmArray BCID panel to excised heart valve tissue. The present study extends these observations to the BCID2 panel and provides additional evidence supporting the feasibility and potential diagnostic utility of this approach.

Direct comparison of diagnostic performance across published studies should, however, be interpreted with caution because of substantial differences in patient populations, specimen processing, molecular platforms, reference standards, and study design. Consequently, variations in reported diagnostic performance are more likely to reflect methodological heterogeneity than intrinsic differences between molecular technologies. Overall, the available evidence indicates that the principal value of molecular testing lies in complementing conventional microbiological diagnostics by increasing pathogen detection in excised heart valve tissue.

In our study, blood culture served as the clinical reference method. Discordant microbiological positivity between blood culture and BCID2 was observed in 10 of 42 patients. In eight cases, blood cultures were positive, whereas BCID2 yielded negative results. *Corynebacterium diphtheriae* and *Lactococcus garvieae* were not detected because they are not included in the BCID2 target panel. In contrast, isolation of *Staphylococcus epidermidis* from blood culture may have resulted from sample contamination during blood collection. For cases involving MSSA, *Streptococcus acidominimus*, or *Enterococcus faecium*, the reason for the lack of BCID2 detection remains unclear. Previous studies have suggested that false-negative valve PCR results may result from sampling a valve fragment that does not contain the infectious focus [5,6].

Differences in microorganism identification between BCID2 and blood culture were also observed. Of particular interest was the detection of *Streptococcus* spp. and *Staphylococcus aureus* by BCID2 despite negative blood cultures for these organisms. This discrepancy may be explained by prior antimicrobial therapy, which eliminated microorganisms from the bloodstream while bacterial DNA from viable or nonviable organisms remained detectable within valve tissue [5].

One noteworthy finding in the present study was the isolation of *Enterococcus faecium* and ESBL-producing *Escherichia coli* from blood cultures, whereas BCID2 identified *Streptococcus* spp. in the corresponding valve specimens. Because non-HACEK Gram-negative bacilli rarely cause infective endocarditis, these discordant results may reflect bloodstream infection unrelated to valve infection, mixed infection, or the effects of antimicrobial therapy before valve excision. Although the available data do not allow determination of the exact explanation, these findings illustrate the complexity of interpreting discordant microbiological results in infective endocarditis. According to published data, *E. coli* accounts for approximately 0.5% of IE cases and is most commonly associated with older women and risk factors such as urinary tract infection, diabetes mellitus, hemodialysis, immunosuppression, and alcohol abuse [12,13,14,15,16].

In one case, MSSA was isolated from blood culture, whereas BCID2 detected both MSSA and *Enterococcus faecalis*. This discrepancy may have resulted from a low burden of *E. faecalis* in the bloodstream and/or insufficient growth in culture media. Furthermore, molecular methods are generally more sensitive than conventional microbiological techniques [17].

Numerous studies have shown that molecular testing of heart valve tissue, particularly in patients with negative blood cultures, may represent an important step toward accelerating the diagnosis of IE [5,10]. However, Marín et al. [9] emphasised that the major limitation of valve-based molecular testing is that it can only be performed in patients who undergo valve surgery.

In the present study, the BCID2 panel was also compared with valve culture. Of the three positive valve culture results, two were consistent with both BCID2 and blood culture findings, while one was considered likely to represent contamination (*Staphylococcus epidermidis* isolated from valve culture, with MSSA identified by both blood culture and BCID2). These findings are consistent with previous reports [17].

In our cohort, *Streptococcus* spp. represented the largest proportion of valve colonisation (38.3%, including *Streptococcus* spp. 34%, *Streptococcus pneumoniae* 2.15%, *Streptococcus agalactia* 2.15%). followed by *Staphylococcus aureus* (19.1%) and *Enterococcus faecalis* (10.6%). Similar findings were reported by Muñoz et al. [18]. In contrast, Murdoch et al. [19], Khan et al. [20] and Patel et al. [21] identified *S. aureus* as the most common cause of IE, followed by *Streptococcus* spp. and *Enterococcus* spp. These differences may reflect variations in study populations and sample sizes.

Several methodological limitations should be considered when interpreting the present findings. First, the study included a relatively small number of surgically treated patients from a single center, limiting the precision of the estimates and the generalizability of the findings. Second, the number of patients with negative blood cultures was limited, which restricted the precision of estimates of specificity and negative predictive value. Third, BCID2 detects only microorganisms included in its predefined panel and therefore cannot identify pathogens outside its analytical scope. Fourth, because blood culture is an imperfect clinical reference method in infective endocarditis, some discordant results may reflect limitations of the comparator rather than of the molecular assay itself study provides further evidence supporting the feasibility of applying the BCID2 assay to excised heart valve specimens as a complementary microbiological diagnostic approach. Fifth, the lack of specific pretreatment data is also a limitation of our study.

## 5. Conclusions

In conclusion, the off-label application of the FilmArray BCID2 assay to excised heart valve specimens appears to be a feasible adjunct to conventional microbiological methods in surgically treated patients with infective endocarditis. Although the assay cannot replace established microbiological techniques, it may provide clinically useful complementary microbiological information, particularly when conventional blood culture and valve cultures are negative. Because of its moderate sensitivity and predefined target panel, negative BCID2 results should not be used to exclude infective endocarditis. Larger prospective multicentre studies are warranted to further define the diagnostic use of this test.

## Figures and Tables

**Table 1:** Identification of infective endocarditis pathogens by different methodological approaches.

Patient	S	Blood Culture Results Before Valve Removal	Valve Type	Multiplex PCR (BCID2 FilmArray) Result	Valve Culture Result
1	M	*Streptococcus gallolyticus*	NVE-mitral	*Streptococcus* spp.	Negative
2	M	*Streptococcus gordoni*	NVE-aortic	*Streptococcus* spp.	Negative
3	M	*Streptococcus mutans*	PVE tricuspid	*Streptococcus* spp.	Negative
4	M	*Streptococcus salivarius*	NVE-aortic	*Streptococcus* spp.	Negative
5	M	*Streptococcus mitis, oralis*	NVE-mitral	*Streptococcus* spp.	Negative
6	M	*Streptococcus gallolyticus*	NVE-aortic	*Streptococcus* spp.	Negative
7	M	*Streptococcus intermedius*	NVE-aortic	*Streptococcus* spp.	Negative
8	F	*Streptococcus parasanguinis*	NVE-aortic	*Streptococcus* spp.	Negative
9	M	*Streptococcus anginosus*	NVE-aortic	*Streptococcus* spp.	No information
10	M	*Streptococcus mutans*	NVE-mitral	*Streptococcus* spp.	Negative
11	M	MSSA	PVE tricuspid	MSSA	MSSE
12	M	MSSA	NVE-tricuspid	MSSA	Negative
13	M	MSSA	PVE aortic	MSSA	Negative
14	F	MSSA	NVE-aortic	MSSA	Negative
15	F	MSSA	NVE-mitral	MSSA	Negative
16	M	MSSA	NVE-tricuspid	MSSA	MSSA
17	M	MSSA	NVE-tricuspid	MSSA	Negative
18	M	MSSA	NVE-tricuspid	MSSA	Negative
19	M	*Enterococcus faecalis*	NVE-aortic	*Enterococcus faecalis*	*Enterococcus faecalis*
			NVE-mitral	*Enterococcus faecalis*	
20	M	*Enterococcus faecalis*	PVE aortic	*Enterococcus faecalis*	Negative
21	M	*Enterococcus faecalis*	PVE aortic	*Enterococcus faecalis*	Negative
22	M	*Enterococcus faecalis*	NVE-aortic	*Enterococcus faecalis*	Negative
23	F	*Enterococcus faecalis*	NVE-aortic	*Enterococcus faecalis*	Negative
24	M	MSSE	NVE-mitral	MSSE	Negative
25	M	MSSE	NVE-tricuspid	MSSE	Negative
26	M	Negative	NVE-tricuspid	Negative	Negative
			NVE-aortic	Negative	
27	F	Negative	PVE aortic, PVE mitral	Negative	Negative
28	M	Negative	aortic	Negative	Negative
29	F	MSSA	NVE-mitral	*E.faecalis*, MSSA	Negative
30	F	*Enterococcus faecium*	NVE-mitral	*Streptococcus* spp.	Negative
31	F	MSSE	NVE-aortic	*Streptococcus agalactiae*	Negative
32	M	*Escherichia coli* ESBL	NVE-mitral	*Streptococcus* spp.	Negative
33	M	Negative	NVE-aortic	*Streptococcus* spp.	Negative
34	M	Negative	NVE-aortic	MSSA	Negative
35	M	*Corynebacterium diphtheriae*	NVE-aortic	Negative	Negative
36	M	MSSA	NVE-mitral	Negative	Negative
37	M	*Streptococcus acidominimus*	NVE-aortic	Negative	Negative
38	M	MRSE	PVE aortic	Negative	Negative
39	M	MSSA, MRSE	NVE-aortic	Negative	Negative
40	M	*Enterococcus faecium*	NVE-tricuspid	Negative	Negative
41	F	*Lactococcus garvieae*	NVE-aortic	Negative	Negative
42	F	MSSA	NVE-mitral	Negative	Negative
43	M	No culture	NVE-aortic	*Streptococcus* spp.	Negative
44	M	No information	NVE-aortic	*Streptococcus* spp.	Negative
45	F	No information	NVE-mitral	*Streptococcus* spp.	Negative
46	M	No information	NVE-aortic	*Streptococcus pneumoniae*	Negative
			NVE-mitral	*Streptococcus pneumoniae*	
47	F	No information	NVE-aortic	Negative	Negative

MSSA—Methicillin-Sensitive *Staphylococcus aureus*; MSSE—Methicillin-Sensitive *Staphylococcus epidermidis*. MRSE—Methicillin-Resistant *Staphylococcus epidermidis*; ESBL—extended-spectrum beta-lactamase; NVE—Native Valve Endocarditis; PVE—Prosthetic Valve Endocarditis.

**Table 2:** Comparison of microbiological positivity between BCID2 and blood culture.

	Blood Culture (+)	Blood Culture (−)
BCID2 (+)	29	2
BCID2 (−)	8	3

**Table 4:** Valve culture vs. multiplex PCR valves.

	BCID2 (+)	BCID2 (−)
Valve culture (+)	3	0
Valve culture (−)	31	12

**Table 5:** Diagnostic performance of valve culture for detection of microbiological positivity compared with BCID2.

95% CI	Estimate	Measure
1.9–23.7%	8.8% (3/34)	Sensitivity
73.5–100.0%	100.0% (12/12)	Specificity
29.2–100.0%	100.0% (3/3)	PPV
15.3–43.7%	27.9% (12/43)	NPV
19.5–48.0%	32.6% (15/46)	Accuracy
−0.011–0.107	0.048	Kappa

## Data Availability

The original contributions presented in this study are included in the article. Further inquiries can be directed to the corresponding author.
